# Supplementary material for: Nutritional status of patients diagnosed with cervical cancer in Tanzania
Source: BMC Nutr. 2026 Jun 2;12:160. doi: 10.1186/s40795-026-01323-2 (PMC13463930; doi:10.1186/s40795-026-01323-2)
Supplement: Supplementary file 1 [file 40795_2026_1323_MOESM2_ESM.docx]

**Consent agreement**:

Hello, I work for the food and nutrition institute here at ORCI. I am coming to you today about your overall health and the food you eat. We know certain foods you eat may be related to how you feel and your recent cervical cancer diagnosis. We want to know what you eat to better understand your diagnosis and help make nutritional recommendations for future patients like you. These questions and activities will take no longer than 20 min. We will collect information on your weight, height, current symptoms, fitness, other diseases, and diet.

**Verbal Consent:** YES	NO,

If **NO** please give reasoning: Lack of time	Too sick	Lack of understanding

Other:

1. **General Intake:**

Patient ID (Medical Number): Date:

Patient Address (City& Region ONLY):

Patients Date of Birth: Ethnicity: Gender (circle one): Male/Female/Other

Patients’ Cervical Cancer Stage (circle one): I	II	III	IV

Patients’ Current Medications:

3. **Food Intake:** As compared to their normal intake, the patient would rate their food intake during the past month as:

Unchanged	More than usual	Less than usual

Talking:

Normal food but less than the normal amount

Little solid food

Only liquids

Only nutritional Supplements

Very little anything

Only tube feedings or nutrition by vein

c
c

2. **Weight:**

Patient current weight: kg

Patient current height: cm

Patients most previous weight:kg

Date of previous weight:kg

During the past two weeks the patients’ weight has:

decreased	not changed	increased

c
c
c

5. **Activities and Function:**

Over the past month, the patient would generally rate their activity as:

normal with no limitations

not my normal self, but able to be up and about with fairly normal activities

not feeling up to most things, but in bed or chair less than half of the day

able to do little activity and spend most of the day in bed or chair

pretty much bed ridden, rarely get out of bed

4. **Symptoms:** The patient has had the following problems that have kept them from eating enough during the past two weeks:

no problem eating

no appetite, just didn’t feel like eating

nausea

constipation

things taste funny or have no taste

smell bothers me

vomiting

diarrhea

mouth sores

problems swallowing

feel full quickly

fatigue

pain: where?

other

c
c
c
c
c
c
c
c
c
c
c
c
c
c
c
c

6. **Disease state/comorbidities** Check all that apply

Cancer	Presence of decubitus, open wound, or fistula	Age greater than 65

AIDS/HIV	HPV	Presence of trauma

Pulmonary or cardiac cachexia	Chronic renal insufficiency

Other relevant diagnoses (specify)

7. **Measuring Metabolic Demand**

Patients’ temperature: Celsius

If at or above 38.8 degrees, duration of patient fever: hours

8. **Measuring Skinfold Thickness**

Chest:	Abdomen	Thigh

1.mm	1.mm	1.mm

2.mm	2.mm	2.mm

3.mm	3.mm	3.mm

Average:mm	Average:mm	Average:mm

Other comments:

The remainder of this document will be completed by the Health Administrator

**Section 1- Scoring weight loss**

To determine score, use 1-month weight data if available. Use 6-month data only if there is no 1-month weight data. Use points below to score weight change and add one extra point if patient has lost weight during the past 2 weeks. Enter total point score in Box 1.

| Weight loss in 1 month | Points | Weight loss in 6 months |
| --- | --- | --- |
| 10% or greater | 4 | 20% or greater |
| 5-9.9% | 3 | 10-19.9% |
| 3-4.9% | 2 | 6-9.9% |
| 2-2.9% | 1 | 2-5.9% |
| 0-1.9% | 0 | 0-1.9% |
| Weight gain in 1 month | **Points** | **Weight gain in 6 months** |
| 10% or greater | 4 | 20% or greater |
| 5-9.9% | 3 | 10-19.9% |
| 3-4.9% | 2 | 6-9.9% |
| 2-2.9% | 1 | 2-5.9% |
| 0-1.9% | 0 | 0-1.9% |

Patients computed BMI:	**Numerical score for section 1**

*Score will be added to boxes 1-4

**Section 2- Disease and its relation to nutritional requirements**

Score is derived by adding 1 point for each of the following conditions:

Cancer	Presence of decubitus, open wound, or fistula	Age greater than 65

AIDS/HIV/HPV	Presence of trauma

Pulmonary or cardiac cachexia	Chronic renal insufficiency

Other relevant diagnoses (specify)

**Numerical score for section 2**

**Section 3- Metabolic Demand**

Score for metabolic stress is determined by several variables known to increase protein & caloric needs. Note: Score fever intensity or duration, whichever is greater. The score is additive so that a patient who has a fever of 38.8 °C (3 points) for < 72 hrs (1 point) and who is on 10 mg of prednisone chronically (2 points) would have an additive score for this section of 5 points.

| Stress | None (0) | Low (1) | Moderate (2) | High (3) |
| --- | --- | --- | --- | --- |
| Fever | No fever | >37.2 and <38.3 | ≥ 38.3 and < 38.8 | ≥ 38.8 °C |
| Fever duration | No fever | <72 hours | 72 hours | > 72 hours |
| Corticosteroids | No corticosteroids | Low does (< 10 mg prednisone equivalents/day) | moderate dose (≥ 10 and < 30 mg prednisone equivalents/day) | high dose (≥ 30 mg prednisone equivalents/day) |

**Numerical score for section 3**

**Section 4 – Physical Exam**

Exam includes a measure of body fat percentage. A skinfold caliper will be used to pull subcutaneous fat away from the muscle measuring the skin-fold thickness. Percent of body fat will depend on age, height, and weight (3 points). This chart is a generality and should be secondary to a chart.

|  | % Of body fat | Points |
| --- | --- | --- |
| Underweight | 15% or below | 3 |
| Lean | 11-26% | 1, 2 (17-24%) |
| Ideal | 17-31% | 0 |
| Average | 23-37% | 1 |
| Over average | 30-39% | 2 |
| Severely Over average | 35-39% or above | 3 |

**Section 5 – Other (*if applicable*)** *This section will not be added to the total score*

**Blood work:**

| WBC: | Vitamin D: |
| --- | --- |
| RBC: | Vitamin B12: |
| Hemoglobin: | Cholesterol: |
| HCT: | Triglyceride: |
| MCV: | HDL Cholesterol: |
| MCH: | LDL Cholesterol: |
| MCHC: | Non-HDL Chol, Calculated: |
| RDW: | Cholesterol/HDL Ratio: |
| Platelets: | Hours Fasting: |
| Ferritin: | Glucose: |

**Liver Function Test:**

Alanine transaminase (ALT):

Aspartate transaminase (AST):

Alkaline phosphatase (ALP):

Albumin and total protein:

Bilirubin:

Gamma-glutamyl transferase (GGT):

L-lactate dehydrogenase (LD):

Prothrombin time (PT):

**Date:**

**Numerical score for section 4**

Total Score:

Global Category Rating:

Nutritional Triage Recommendation:

Healthcare Professional Signature:

Position Title: Date:

**Total Score (Total numerical score of A+B+C+D):**

**Global Category rating (Stage A, Stage B, or Stage C):**

|  | **Stage A** | **Stage B** | **Stage C** |
| --- | --- | --- | --- |
|  | Well-nourished | Moderate/suspected malnutrition | Severely malnourished |
| **Category Weight** | No weight loss or gain | ≤ 5% loss in 1 month (≤10% in 6 months) OR progressive weight loss | > 5% loss in 1 month (>10% in 6 months) OR progressive weight loss |
| **Nutrient intake** | No deficit OR Significant recent improvement | Definite decrease in intake | Severe deficit in intake |
| **Nutrition Impact Symptoms (NIS)** | None OR significant recent improvement allowing adequate intake | Presence of NIS (BOX 3) | Presence of NIS (BOX 3) |
| **Functioning** | No deficit OR Significant recent improvement | Moderate functional deficit OR Recent deterioration | Severe functional deficit OR Recent significant deterioration |
| **Physical Exam** | No deficit OR chronic deficit | Evidence of mild to moderate increase of decrease of body fat percentage | Severe functional deficit |

**Nutritional Triage Recommendations:** Additive score is used to define specific nutritional interventions including patient & family education, symptom management including pharmacologic intervention, and appropriate nutrient intervention (food, nutritional supplements, enteral, or parenteral triage).

***First line nutrition intervention includes optimal symptom management.***

**Triage based on point score**

- **0-1:** No intervention required at this time. Re- assessment on routine and regular basis during treatment.
- **2-3:** Patient & family education by dietitian, nurse, or other clinician with pharmacologic intervention as indicated by symptom survey (Box 3) and lab values as appropriate.
- **4-8:** Requires intervention by dietitian, in conjunction with nurse or physician as indicated by symptoms (Box 3).
- **≥ 9:** Indicates a critical need for improved symptom management and/or nutrient intervention options.

*This patient generated nutritional assessment form is derived from the Scored Patient-Generated Subjective Global Assessment (PG-SGA).

Source: PG-SGA©. (2014, March 27). *Pt-Global*. https://pt-global.org/pt-global/
