# Supplementary material for: Nutritional status of patients diagnosed with cervical cancer in Tanzania
Source: BMC Nutr. 2026 Jun 2;12:160. doi: 10.1186/s40795-026-01323-2 (PMC13463930; doi:10.1186/s40795-026-01323-2)
Supplement: Supplementary file 2 [file 40795_2026_1323_MOESM3_ESM.docx]

**Social Participant History**

Finally, I am going to ask the following questions to help us better understand you and their current situation.

Please check the boxes that best represent the participant’s current status

| Do you smoke cigarettes? | Never Yes #Packs a day  Quit Date quit Years smoked |
| --- | --- |
| Do you vape (e-cigarette)? | No Yes |
| Do you drink alcohol? | Never Yes # Drinks per week |
| What is your highest level of education completed? | High School Trade School College Post-graduate |
| Are you employed? | No Retired Yes Type of work |
| Do you exercise? | No Yes Type  How often How long per activity |
| Are you sexually active? | No Yes # of sexual partners  Men Women Both |
| What is your marital status | Single Married Widowed Separated Divorced |
| Do you have children? | No Yes If yes # of children  v |
| How many persons are currently living in your home? |  |
| Family history of cancer? | No Yes |

**Health Screening**

| *Housing Utilities* |  |
| --- | --- |
| Within the past 6 months, have you ever stayed: outside, in a car, in a tent, in an overnight shelter, or temporarily in someone else house? |  |
| Are you worried about losing your house? |  |
| Within the past 6 months, have you been unable to get utilities (heat, electricity) when it was really needed? |  |
| *Transportation* |  |
| Within the past 6 months, has a lack of transportation kept you from medical appointments or from doing things needed for daily living? |  |
| *Interpersonal Safety* |  |
| Do you feel physically or emotionally safe where you live? |  |
| *Healthcare* |  |
| Within the past 6 months, have you decided not to purchase medications because they were too expensive? |  |

| *Food* |  |
| --- | --- |
| Within the past 6 months, did you worry the food would run out before you got money to buy more? |  |

| *HPV* |  |
| --- | --- |
| Have you heard of the HPV vaccine? |  |
| Do you have the HPV vaccine? |  |
| Would/do your friends and family support you if you were to get/got vaccinated? |  |
| Are you worried about the cost of the HPV vaccine? |  |
| Are you concerned about the side effects of the HPV vaccine? |  |

**Please list any concerns the participant has with the HPV vaccine:**

Time interview ended:

Participant Signature:

Healthcare Professional Print Name:

Healthcare Professional Signature:
