# Supplementary material for: Nutritional status of patients diagnosed with cervical cancer in Tanzania
Source: BMC Nutr. 2026 Jun 2;12:160. doi: 10.1186/s40795-026-01323-2 (PMC13463930; doi:10.1186/s40795-026-01323-2)
Supplement: Supplementary file 3 [file 40795_2026_1323_MOESM1_ESM.doc]

 Now, I will ask you the describe the foods (meals and snacks) that you ate yesterday during the day and night, whether at home or outside the home. Start with the first food eaten in the morning*.*

|  | **Individual Dietary Diversity (IDD)** | Score | Frequency of consumption | | |
| --- | --- | --- | --- | --- | --- |
|  | **From the list of foods consumed in the 24-hour dietary recall above,** score 1 if any of the food items within a food group was consumed and 0 if the food item was not consumed in the past 24 hours, and then ask for the frequency of consumption | 1=Yes  0=No | Per week | Per month | Per year |
| Cereals | a) Any *(*Bread, rice, or other foods made from grains, oats, maize, barley, wheat, sorghum, millet or other grains? *(Other locally available grain e.g ugali, porridge)*? |  |  |  |  |
| Vitamin A rich vegetables and tubers | Pumpkin, carrots, squash, or sweet potatoes that are orange inside + *other locally available vitamin-A rich vegetables(e.g.*  *sweet pepper)* |  |  |  |  |
| White tubers and  Roots | White potatoes, white yams, cassava, or foods made from roots. |  |  |  |  |
| Dark green leafy  Vegetables | Dark green/leafy vegetables, including wild ones + *locally available vitamin-A rich leaves such as cassava leaves etc.* |  |  |  |  |
| Other vegetables | Other vegetables (e.g. tomato, onion, eggplant) , including wild vegetables |  |  |  |  |
| Vitamin A rich fruits | Ripe mangoes, cantaloupe, dried apricots, dried peaches + *other locally available vitamin A-rich fruits* |  |  |  |  |
| Other fruits | Other fruits, including wild fruits |  |  |  |  |
| Organ meat (iron rich) | Liver, kidney, heart or other organ meats or blood-based foods |  |  |  |  |
| Flesh meats | Beef, pork, lamb, goat, rabbit, wild game, chicken, duck, or  other birds |  |  |  |  |
| Eggs |  |  |  |  |  |
| Fish | Fresh or dried fish or shellfish |  |  |  |  |
| Legumes, nuts and  Seeds | Beans, peas, lentils, nuts, seeds or foods made from these |  |  |  |  |
| Milk and milk  Products | Milk, cheese, yogurt or other milk products |  |  |  |  |
| Oils and fats | Oil, fats or butter added to food or used for cooking |  |  |  |  |
| Sweets | Sugar, honey, sweetened soda or sugary foods such as chocolates, sweets or candies |  |  |  |  |
| Spices, condiments, beverages | Spices (black pepper, salt), condiments (soy sauce, hot sauce), coffee, tea, alcoholic beverages OR *local examples* |  |  |  |  |
|  | | | | | YES=1  NO=0 |
| Individual  level only | Did you eat anything (a meal or snack) OUTSIDE of the home yesterday? | | | |  |
